# Partitioned RIS-Assisted Vehicular Secure Communication Based on Meta-Learning and Reinforcement Learning

**DOI:** 10.3390/s25185874

**Published:** 2025-09-19

**Authors:** Hui Li, Fengshuan Wang, Jin Qian, Pengcheng Zhu, Aiping Zhou

**Affiliations:** 1 College of Information Engineering, Taizhou University, Taizhou 225300, China; huili_tzxy@tzu.edu.cn (H.L.); zhupcnt@tzu.edu.cn (P.Z.); zhouaiping@tzu.edu.cn (A.Z.); 2 School of Computer Science and Engineering, Xi’an University of Technology, Xi’an 710048, China; fswang@stu.xaut.edu.cn

**Keywords:** vehicular ad hoc networks, physical layer security, reconfigurable intelligent surface, reinforcement learning, meta learning

## Abstract

This study tackles the issue of ensuring secure communications in vehicular ad hoc networks (VANETs) under dynamic eavesdropping threats, where eavesdroppers adaptively reposition to intercept transmissions. We introduce a scheme utilizing a partitioned reconfigurable intelligent surface (RIS) to assist in the joint transmission of confidential signals and artificial noise (AN) from a source station. The RIS is divided into segments: one enhances legitimate signal reflection toward the intended vehicular receiver, while the other directs AN toward eavesdroppers to degrade their reception. To maximize secrecy performance in rapidly changing environments, we introduce a joint optimization framework integrating meta-learning for RIS partitioning and reinforcement learning (RL) for reflection matrix optimization. The meta-learning component rapidly determines the optimal RIS partitioning ratio when encountering new eavesdropping scenarios, leveraging prior experience to adapt with minimal data. Subsequently, RL is employed to dynamically optimize both beamforming vectors as well as RIS reflection coefficients, thereby further improving the security performance. Extensive simulations demonstrate that the suggested approach attain a 28% higher secrecy rate relative to conventional RIS-assisted techniques, along with more rapid convergence compared to traditional deep learning approaches. This framework successfully balances signal enhancement with jamming interference, guaranteeing robust and energy-efficient security in highly dynamic vehicular settings.

## 1. Introduction

### 1.1. Background

The swift evolution of intelligent transportation systems (ITS) has established wireless VANETs as a pivotal technology for the future, facilitating essential services that span from preventing collisions to enhancing traffic efficiency optimization [1,2,3,4]. However, the open broadcast nature of wireless channels in high-mobility vehicular environments renders communications exceptionally vulnerable to eavesdropping attacks, where malicious actors can intercept sensitive data such as vehicle trajectories, safety messages, and user identities [5]. Traditional cryptographic security mechanisms, while foundational, introduce prohibitive latency and computational overhead that undermine real-time safety-critical operations in dynamic VANET scenarios [6]. To overcome these limitations, physical layer security (PLS) has arisen to be a complementary paradigm, exploiting inherent channel properties such as fading, noise, and interference to inherently degrade eavesdropper reception without additional encryption delays [7]. Nevertheless, the ultra-dynamic topology of VANETs, characterized by rapidly changing line-of-sight conditions and mobile eavesdroppers capable of adaptive repositioning, demands highly responsive and resource-efficient security solutions beyond conventional static approaches, yet remains largely unexplored for VANET-specific security applications [8].

Among various PLS techniques, artificial noise generation and cooperative jamming are two widely adopted strategies for safeguarding confidential transmissions [9,10]. AN involves transmitting deliberately crafted interference signals alongside legitimate data to deteriorate Eve’s decoding capability, whilst having little effect on the intended recipient, who can mitigate the noise through spatial separation or prior channel knowledge [11]. In contrast, cooperative jamming utilizes trusted relays or helper nodes to emit jamming signals that obscure the confidential message from potential eavesdroppers [12]. These helper nodes may operate either in coordination with the legitimate transmitter or independently, depending on the network topology. Both techniques are particularly effective in multi-antenna and distributed network settings, where spatial degrees of freedom can be leveraged [13].

Reconfigurable intelligent surface assisted wireless communication has emerged as a transformative enabler in the evolution toward 6G networks [14,15]. Unlike conventional active relays, a RIS comprises numerous inexpensive, passive elements capable of adaptively adjusting both amplitude and phase when receiving electromagnetic signals. Through clever configuration of these components, RIS allows real-time control over the radio transmission surroundings, enhancing the reception, expanding the coverage, and mitigating interference [16]. This paradigm transforms the radio environment from a passive medium into an active asset for communication optimization. Easily deployable on existing infrastructure, such as building facades or indoor surfaces, RIS improves both spectral and energy efficiency with minimal power consumption and without introducing additional noise. Its inherent flexibility also makes it highly suitable for dynamic applications in unmanned aerial vehicles (UAVs), the Internet of Things (IoT), as well as secure communications [17]. As research progresses, RIS-assisted systems are poised to become foundational to underpin the creation of smart, programmable, and sustainable network infrastructures.

This investigation addresses distributed constrained online convex optimization (OCO) in multi-agent systems that communicate over fading multiple access channels, with the objective of minimizing the cumulative global loss subject to overall constraints [18]. A separate joint optimization framework is also presented for IRS-supported MISO systems, aiming to enhance sum-rate through the coordinated design in IRS-user pairings, source station beamforming, as well as IRS phase shifts. To resolve this non-convex mixed-integer optimization challenge, a convex relaxation technique is utilized that decomposes it into three subproblems solved iteratively. Specifically, the binary variables for IRS-user association are first relaxed into a continuous [0,1] interval, transforming the subproblem to convex form that is subsequently addressed through a duality based solution approach, KKT conditions, fixed-point iteration, and gradient descent [19].

Deep reinforcement learning (DRL) synergistically mixtures illustrative power in deep learning with a making decisions framework of reinforcement learning, facilitating agents to learn complicated control policies in high-dimensional, dynamic environments [20,21]. DRL has achieved notable success in domains like strategic gameplay (e.g., AlphaGo), autonomous driving, and robotic control. In the field of wireless communications, DRL is increasingly utilized for tasks such as dynamic spectrum access, power allocation, resource scheduling, and secure transmission, especially in scenarios characterized by partial observability and environmental uncertainty [22]. Methods that facilitate both model-free and model-based learning include Deep Q-Networks, Policy Gradient approaches, and Actor–Critic architectures, making them well-suited for real-time adaptive decision-making [23]. To further enhance DRL’s practicality, emerging innovations like experience replay, target networks, and meta-learning are being integrated to improve stability, sample efficiency, and exploration strategies, reinforcing DRL’s role as a core enabler of intelligent wireless systems.

This study examines the capacity of partitioned RIS systems, which utilize meta-learning and reinforcement learning, to swiftly adjust to novel environmental conditions in dynamic settings and to enhance communication security. The presented methodology is implemented in VANETs, yet remains suitable for broader applications. Its adaptability to changing environments is thereby demonstrated.

### 1.2. Motivation

Previous studies on intermittent interference have revealed that when a jamming signal’s strength surpasses specific thresholds, it targets eavesdroppers; further increases in interference intensity yield minimal improvements in system security performance [24,25,26]. This indicates the existence of a saturation point beyond which additional jamming becomes inefficient. Therefore, identifying an appropriate interference power range is crucial, not only to effectively safeguard the system against eavesdropping threats but also to reduce overall energy consumption, thereby enhancing the energy efficiency of secure communication systems.

As noted in study [27,28], meta-learning enables models to achieve satisfactory performance on fresh assignments with minimal data by leveraging prior experience gained across multiple related tasks. This characteristic is especially beneficial for communication systems, for which most data contains sensitive user information, limiting the availability of large-scale training datasets for new environments. Meta-learning effectively addresses the challenge of data scarcity in such scenarios. Moreover, its high convergence efficiency makes it well-suited for dynamically changing environments, where rapid adaptation is essential.

In earlier wireless secure communication systems assisted by RIS partitioning, partitioning ratios were typically determined through convex optimization techniques [29]. However, in dynamic eavesdropping environments, the high mathematical complexity of conventional convex optimization methods often hinders their practical implementation and limits the potential for further improvements in system security performance. Prior work has investigated the separate use of reinforcement learning (RL) and meta-learning for RIS-assisted communications [28,30]. However, the deep integration of a meta-learning module to achieve rapid RIS partitioning ratio adaptation against a mobile eavesdropper, combined with a MADDPG-based reflection optimizer in a partitioned RIS system, presents an unresolved challenge for vehicular networks. Building upon this foundation, we introduce a novel integrative framework to address this gap. Our incremental but significant contribution lies in the synergistic combination of these advanced techniques, resulting in a highly responsive security solution designed for the ultra-dynamic VANET environment with its rapidly shifting line-of-sight conditions and adaptively moving eavesdroppers.

### 1.3. Our Method and Main Contributions

In order to fill in the above described lack of research, this work proposes a unique approach that combines reinforcement learning-based RIS reflection matrix optimization with meta-learning based RIS partitioning method. The objective is to improve reception of the authorized receiver while intensifying impact of AN on eavesdroppers, thereby significantly improving the secure communication performance of the system. Specifically, in a dynamic eavesdropping environment, meta-learning is employed to determine the optimal RIS partitioning ratio, dividing the RIS into segments responsible for reflecting legitimate signals and for reinforcing AN effects. Subsequently, reinforcement learning is utilized to optimize RIS’s reflective matrix coefficient, for the intention of increasing the network’s secrecy rate. The principal contributions of this paper are summarized as follows:

- We investigate a partitioned RIS aided wireless VANET, where the source station transmits both confidential messages and AN. The partitioned RIS elements are configured to reflect the legitimate signals toward the intended vehicular receiver and direct the AN toward the eavesdropper. The wireless communication network’s overall secrecy performance is strengthened by this dual-reflection technique, which improves the signal strength for the authorized vehicular user while amplifying the AN’s disruptive effect on eavesdropper.
- We propose a secure communication scheme that integrates a meta-learning based partitioning method with reinforcement learning based optimization of the RIS reflection matrix for dynamic eavesdropping environments. Specifically, when encountering a new eavesdropping scenario, the meta-learning model rapidly determines the optimal RIS partitioning ratio to balance the reflection of legitimate signals and artificial noise. Subsequently, reinforcement learning is utilized to optimize beamforming as well as RIS reflection matrixes, thereby making the wireless communication network as secure as possible.
- We conduct comprehensive simulation experiments to confirm the suggested scheme’s efficacy in enhancing communication security under dynamic eavesdropping conditions. Compared to traditional RIS-assisted wireless systems, our approach exhibits significantly improved secure communication performance. Moreover, the meta-learning based partitioning method demonstrates faster convergence than conventional deep learning techniques, enabling better adaptability to rapidly changing eavesdropping environments.

### 1.4. Related Work

Numerous studies have been conducted on PLS as a complementary approach to traditional cryptographic techniques for securing wireless communications [31,32,33]. A reconfigurable intelligent surface approach enables dynamic control of the wireless environment, enhancing signal quality and suppressing eavesdropping in secure communication systems [34,35,36]. Deep reinforcement learning enables adaptive policy learning for intelligent resource allocation and interference management, improving robustness and secrecy in wireless communication networks [30,37,38].

PLS ensures protection by taking advantage of the intrinsic characteristics of radio channels, providing an efficient as well as low-complexity security approach for wireless communication systems without dependence on conventional key management [31,32,33]. For collaborative cognitive radio networks, to increase PLS performance, Wen et al. suggested a secret jamming technique that targets an intelligent eavesdropper (Eve) [31]. To enhance communication security, a backscatter device (BD) and a quadratic capture of energy model are utilized, which cooperate with a decode-and-forward (DF) relay [32]. Su et al. study combined sensing and communication networks’ sensing-assisted physical layer security (PLS) [33].

RIS technology improves wireless security by actively shaping the radio environment to boost the desired signal at legitimate receivers while reducing the risk of interception, thus enhancing the physical layer defense in communication networks [34,35,36]. A methodical methodology was developed by Saggese et al. to assess how management actions impact communication efficiency across data channel rate decision dimensions and control channel bandwidth allocation [34]. A precise analysis of RIS-assisted vehicular communication with coherent combining of RIS-reflected and direct signals under uniformly distributed phase errors and generalized fading was reported by Chapala et al. [35]. A filtering RIS with 2-bit phase-shifting and strong frequency selection was proposed by Liang et al. [36].

By enabling intelligent decision-making in complex and dynamic wireless environments, DRL enhances capacity for secure communication networks in optimizing resource allocation and counteracts potential security threats in real time [30,37,38]. With the use of a reconfigurable intelligent surface, Chen et al. examines downlink orthogonal frequency division multiplexing distribution network with the intention of enhancing spectrum effectiveness and communication reliability through clever channel management and reflection [30]. Aung et al. propose a mechanism for downlink communication among the users and the base station, aided by several active reconfigurable intelligent surfaces (ARISs) [37]. In order to determine whether a wireless device (WD) should perform local computation or offload the task to one of the high-altitude platforms (HAPs), Zhang et al. introduced a DRL framework utilizing online deep neural networks to produce almost ideal decisions for resource dumping [38].

As wireless networks evolve toward massive connectivity and high mobility, integrating artificial noise and cooperative jamming with reconfigurable intelligent surfaces and learning-based algorithms is expected to be crucial for achieving scalable and resilient secure communications.

## 2. System Model

In practical vehicular scenarios, the mobility of both the intended receiver (D) and an eavesdropper (E) introduces time-varying channels. To reconcile model fidelity with computational tractability, this work employs a “block-fading” channel model for VANETs. This approach discretizes the continuous communication timeline into distinct frames. During each frame, corresponding to one signal transmission, the channel is treated as static, justified by the orders-of-magnitude difference between the channel’s coherence time and the data symbol period. The channel state is updated only at the transition between frames, reflecting new positional information as vehicles move. By ensuring each frame duration is substantially shorter than channel coherence time, this model effectively approximates the time-varying nature of mobile vehicular channels, thereby supporting the feasibility of high-speed communications.

### 2.1. Signal Transmission Model

**Figure 1:**
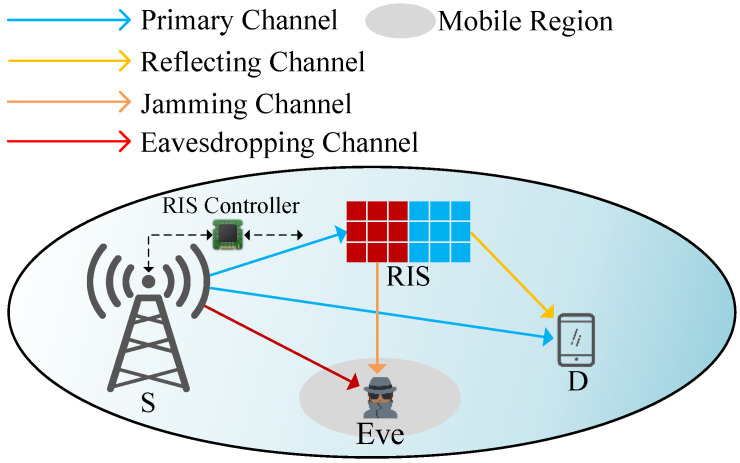
System model.

As depicted in Figure 1, we investigate RIS-aided secure VANETs with a multiple-antennas source station, a single-antenna signal transmission destination, a RIS consisting of multiple reflecting elements, and an intelligent eavesdropper. The source station, signal transmission destination, and intelligent eavesdropper are represented as *S*, *D*, and *E*, respectively. To offer broadcast variety for the confidential information and AN, the *S* is outfitted with two distinct antennas $N_{s}$, both of which have complicated normal distributions. Confidential information *m* is transmitted by the first antenna ($N_{m}$), and noise *n* is conveyed by the second ($N_{n}$) to improve the interference impact at *E*. The total transmit power at *S* is $P_{s}\geq P_{m}+P_{n}$, where $P_{m}$ and $P_{n}$ are corresponding power distributions to *m* and *n*. RIS has $N_{r}$ reflective components. The *S* transmits its confidential information to *D*, while the RIS receives legitimate signals and interference signals from *S* and sends them to *D* and *E*, respectively.

Specifically, when *S* transmits its confidential information to *D*, we divide the reflective elements of the RIS into legitimate signal collaboration reflection areas and artificial noise reflection areas, and by adjusting the reflection coefficient at RIS, we broadcast different messages toward *D* and *E*, respectively. Therefore, RIS improves signal-to-noise ratio at *D* by reflecting confidential messages to *D*, whilst intensifying artificial noise’s effect at *E* by reflecting artificial noise to *E*, thereby ensuring communication security of the wireless system. In addition, when the intelligent eavesdropper is unable to effectively eavesdrop on signals due to interference or obstruction, it will change position to improve its eavesdropping capabilities.

The channel coefficients from *S* to RIS, *E*, *D*, from RIS to *E*, *D* are expressed as $h_{sr},h_{se}$, $h_{sd}$, $h_{re},h_{rd}$, respectively; s∈{m,n}. Specifically, $h_{sr}\in C^{N_{r}\;\times \;N_{s}}$, $h_{se}\in C^{1\;\times \;N_{s}}$, $h_{sd}\in C^{1\;\times \;N_{s}}$, $h_{re}\in C^{1\;\times \;N_{r}}$, $h_{rd}\in C^{1\;\times \;N_{r}}$ are represented as the channel vector from *S* to RIS, *E*, *D*, from RIS to *E*, respectively; s∈{m,n}. Moreover, the Euclidean distances from *S* to RIS, *E*, *D*, from RIS to *E*, *D* are denoted as $d_{sr}$, $d_{se}$, $d_{sd}$, $d_{re}$, $d_{rd}$, respectively. In addition, $\sigma_{d}^{2}$ and $\sigma_{e}^{2}$ are additive white Gaussian noise (AWGN) at *D* and *E*, respectively.

The *S* employs beamforming vector $w_{m}$ and $w_{n}$ to transmits confidential information signal and artificial noise, assuming that channels follow the flat-fading, quasi-static channel paradigm. The source station *S* transmit L={1,⋯,L} signal symbols, where signal s(l)∼CN(0,1), with l∈L. The RIS manipulates programmable reflecting elements by a reflection matrix Φ, expressed as

$$\Phi =\mathrm{diag}(\beta_{1}e^{j\theta_{1}},\beta_{2}e^{j\theta_{2}},\cdots ,\beta_{N_{r}}e^{j\theta_{N_{r}}}),$$

where $\beta_{n}\in \left[0,1\right]$ and $\theta_{n}\in \left[0,2\pi \right)$ symbolize the *n*th RIS component’s controllable amplitude and phase shifts.

In this research, a partitioned RIS approach is proposed to ensure communication reliability. The RIS components are allocated to individual users using a partitioning factor α∈[0,1]. The first $\left\lfloor \alpha N_{r}\right\rfloor$ components are specifically allocated to direct the confidential signals toward *D*. Their configuration is described by the matrix $\Phi_{d}$, derived from Φ by setting the last $N_{r}-\left\lfloor \alpha N_{r}\right\rfloor$ elements to zero:

$$\Phi_{d}=\mathrm{diag}\left(\beta_{1}e^{j\theta_{1}},\cdots ,\beta_{\left\lfloor \alpha N_{r}\right\rfloor }e^{j\theta_{\left\lfloor \alpha N_{r}\right\rfloor }},0,\cdots ,0\right).$$

Conversely, the remaining $\left\lceil \left(1-\alpha \right)N_{r}\right\rceil$ elements are dedicated to directing AN toward *E*. Their configuration is described by the matrix $\Phi_{e}$, derived from Φ by setting the first $\left\lfloor \alpha N_{r}\right\rfloor$ elements to zero:

$$\Phi_{e}=\mathrm{diag}\left(0,\cdots ,0,\beta_{\lfloor \alpha N_{r}\rfloor +1}e^{j\theta_{\lfloor \alpha N_{r}\rfloor +1}},\cdots ,\beta_{N_{r}}e^{j\theta_{N_{r}}}\right).$$

This ensures $\Phi =\Phi_{d}+\Phi_{e}$.

### 2.2. Secrecy Rate Maximization Formulation

Following the derivation of the secrecy performance statement, we formulate optimization problems under constraints in this section.

#### 2.2.1. Secrecy Rate

A common theoretical premise in the study of artificial noise (AN)-assisted PLS is perfect cancellation of the AN component by the legitimate receiver *D* [11]. This assumption, which establishes an upper bound for potential secrecy performance, rests fundamentally on two principal techniques.

Beamforming vector $w_{n}$ for the artificial noise is spatially precoded to reside within the null space of *D*’s composite channel. Given perfect channel state information (CSI), this guarantees the AN component is orthogonal to the legitimate receiver’s channel, thus causing no interference.

Prior Knowledge: If the artificial noise is generated from a pseudo-random sequence known only to *S* and *D*, then *D* can leverage this prior knowledge to coherently subtract the known interference from its received signal $y_{d}\left(l\right)$.

Under this assumption, with beamforming vector $w_{m}$ and $w_{n}$, reflection matrixes $\Phi_{d}$ and $\Phi_{e}$, transmission signals that *D* and *E* obtained can be represented as

(1) $$\begin{array}{cc} y_{d}\left(l\right) & =\left(h_{md}+h_{mr}\alpha \Phi_{d}h_{rd}+h_{mr}\Phi_{e}h_{rd}\right)w_{m}m\left(l\right)\\ \; & +\left(h_{nd}+h_{nr}\alpha \Phi_{d}h_{rd}+h_{nr}\Phi_{e}h_{rd}\right)w_{n}n\left(l\right)\\ \; & +\sigma_{d}^{2}, \end{array}$$

(2) $$\begin{array}{cc} y_{e}\left(l\right) & =\left(h_{me}+h_{mr}\alpha \Phi_{d}h_{re}+h_{mr}\Phi_{e}h_{re}\right)w_{m}m\left(l\right)\\ \; & +\left(h_{ne}+h_{nr}\alpha \Phi_{d}h_{re}+h_{nr}\Phi_{e}h_{re}\right)w_{n}n\left(l\right)\\ \; & +\sigma_{e}^{2}, \end{array}$$

where $\sigma_{d}^{2}$ and $\sigma_{e}^{2}$ are AWGN of *D* and *E*, following distribution $\sigma_{d}^{2}∼\mathrm{CN}\left(0,\sigma_{d}^{2}\right)$, $\sigma_{e}^{2}∼\mathrm{CN}\left(0,\sigma_{e}^{2}\right)$. If *D* is aware of artificial noise, it is able to eliminate it from the signal that was transmitted. The signal-to-noise ratio (SNR) at the *D* can be expressed as

(3) $$\begin{array}{c} \gamma_{d}=\frac{W_{m}{\left|h_{md}+h_{mr}\alpha \Phi_{d}h_{rd}+h_{mr}\Phi_{e}h_{rd}\right|}^{2}}{\sigma_{d}^{2}}, \end{array}$$

where $W_{m}=w_{m}w_{m}^{H}$. In contrast, *E* fails to distinguish AN, misinterpreting it as interference. Accordingly, signal-to-interference-and-noise ratio (SINR) for *E* is represented by

(4) $$\begin{array}{c} \gamma_{e}=\frac{W_{m}{\left|h_{me}+h_{mr}\alpha \Phi_{d}h_{re}+h_{mr}\Phi_{e}h_{re}\right|}^{2}}{W_{n}{\left|h_{ne}+h_{nr}\alpha \Phi_{d}h_{re}+h_{nr}\Phi_{e}h_{re}\right|}^{2}+\sigma_{e}^{2}}, \end{array}$$

where $W_{n}=w_{n}w_{n}^{H}$. The beamforming vectors for the confidential message and artificial noise are represented by the lowercase bold symbols $w_{m}$ and $w_{n}$, respectively. Their corresponding covariance matrices, denoted by the uppercase bold $W_{m}$ and $W_{n}$, are defined as $W_{m}=w_{m}w_{m}^{H}$ and $W_{n}=w_{n}w_{n}^{H}$. These positive semidefinite matrices are employed in SNR and SINR formulations to simplify the optimization process.

Therefore, the secrecy rate at *D* and *E* could be stated as follows:

(5) $$\begin{array}{cc} R_{d} & =\log_{2}\left(1+\gamma_{d}\right), \end{array}$$

(6) $$\begin{array}{cc} R_{e} & =\log_{2}\left(1+\gamma_{e}\right). \end{array}$$

An RIS-assisted secure communication system’s secrecy rate is the distinction among *D* and *E*’s attainable rates. This can be expressed as

(7) $$\begin{array}{c} R_{d,e}=\max\left(R_{d}-R_{e},0\right). \end{array}$$

#### 2.2.2. Secrecy Rate Maximization

The objective of the research is to achieve a maximum secrecy rate jointly optimizing transmitting beamforming $W_{m}$, $W_{n}$, partitioning factor α, and corresponding reflection matrix $\Phi_{d}$, $\Phi_{e}$, subjected to transmission capacity of the source station and RIS’s elements number. The objective of maximizing the secrecy rate can be formulated as the following optimization problem:

(8) $$\begin{array}{cc} P1:\max_{W_{m},W_{n},\alpha ,\Phi_{d},\Phi_{e}} & R_{d,e} \end{array}$$

(9) $$\begin{array}{cc} s.t. & \mathrm{Tr}\left(W_{m}\right)+\mathrm{Tr}\left(W_{n}\right)\leq P_{s}^{m}, \end{array}$$

(10) $$\;0\leq \mathrm{Tr}\left(W_{m}\right)\leq P_{s}^{m},$$

(11) $$\;0\leq \mathrm{Tr}\left(W_{n}\right)\leq P_{s}^{m},$$

(12) 0≤α≤1,

(13) $$\;\mathrm{rank}(W_{m})=1,$$

(14) $$\;\mathrm{rank}(W_{n})=1,$$

where $P_{s}^{m}$ represents the maximum transmission power of the source station.

The non-convex rank-one constraint Equations (13) and (14) imposed on the covariance matrices $W_{m}$ and $W_{n}$ in problem P1 present a significant computational challenge. To overcome this, we utilize semidefinite relaxation (SDR), a well-established method that removes these constraints to form a convex semidefinite program (SDP) tractable by standard solvers. Following the acquisition of the optimal matrices $W_{m}^{*}$ and $W_{n}^{*}$ from the relaxed problem, we apply a Gaussian randomization technique. This procedure generates numerous random vectors from a complex Gaussian distribution defined by these covariance matrices, from which the best candidate satisfying the power constraints and maximizing the objective is selected. While not guaranteed to be globally optimal, this approach is a proven and effective strategy for obtaining high-quality, feasible rank-one approximations, which are essential for the subsequent stages of our meta-learning and reinforcement learning framework.

#### 2.2.3. Optimization of Beamforming Vectors via Semidefinite Relaxation

The beamforming vectors $w_{m}$ and $w_{n}$ are essential to maximize the secrecy rate, as formulated in optimization problem P1. This problem becomes NP-hard due to the non-convex rank constraints $\mathrm{rank}(W_{m})=1$ and $\mathrm{rank}(W_{n})=1$ specified in Equations (13) and (14). To tackle this computational challenge, we utilize semidefinite relaxation (SDR), a well-established method documented in [39].

The SDR procedure is implemented through the following specific steps:

(1) Problem Reformulation: The beamforming vectors are substituted with their respective covariance matrices, defined as $W_{m}=w_{m}w_{m}^{H}$ and $W_{n}=w_{n}w_{n}^{H}$. By construction, these matrices are positive semidefinite (PSD), satisfying $W_{m}⪰0$ and $W_{n}⪰0$. Consequently, the received signal power and interference components within the SNR and SINR formulations (3) and (4) are rewritten as quadratic expressions utilizing these matrices. For example, the term $|hw_{m}{|}^{2}$ is transformed into $\mathrm{Tr}(HW_{m})$, with $H=h^{H}h$.

(2) Relaxation: The original non-convex constraints (13) and (14), which enforce rank-one conditions, are eliminated. This relaxation converts the problem to a convex semidefinite program (SDP) that could be successfully resolved to global optimality using standard interior-point methods available in convex optimization suites like CVX.

(3) Solution Extraction via Gaussian Randomization: The optimal solutions $W_{m}^{*}$ and $W_{n}^{*}$ obtained from the relaxed semidefinite program may not satisfy the rank-one condition. To generate viable beamforming vectors, a Gaussian randomization procedure is subsequently employed.

(4) For *L* randomization trials, we generate candidate vectors: ${\tilde{w}}_{m}^{\left(l\right)}∼\mathrm{CN}\left(0,W_{m}\right),{\tilde{w}}_{n}^{\left(l\right)}∼\mathrm{CN}\left(0,W_{n}\right),l=1,2,\ldots ,L.$

(5) For each candidate pair $\left({\tilde{w}}_{m}^{\left(l\right)},{\tilde{w}}_{n}^{\left(l\right)}\right)$, we perform power scaling to satisfy the total power constraint (9): $w_{m}^{\left(l\right)}=\sqrt{p_{m}^{\left(l\right)}}\frac{{\tilde{w}}_{m}^{\left(l\right)}}{\left|\right|{\tilde{w}}_{m}^{\left(l\right)}\left|\right|},\;w_{n}^{\left(l\right)}=\sqrt{p_{n}^{\left(l\right)}}\frac{{\tilde{w}}_{n}^{\left(l\right)}}{\left|\right|{\tilde{w}}_{n}^{\left(l\right)}\left|\right|},$ where $p_{m}^{\left(l\right)}+p_{n}^{\left(l\right)}\leq P_{s}^{m}$. The power allocation between the message and noise for each candidate can be optimized subject to the constraints. We evaluate the achievable secrecy rate $R_{d,e}^{\left(l\right)}$ for each feasible candidate.

(6) We select the candidate pair $\left(w_{m},w_{n}\right)$ that yields the maximum secrecy rate: $\left(w_{m},w_{n}\right)=\underset{l\in 1,\ldots ,L}{\arg\max}\;R_{d,e}^{\left(l\right)}.$

This SDR-based approach provides a high-performance benchmark for the beamforming design.

### 2.3. Beamforming and Partitioned Optimization Based on Meta-Learning and Reinforcement Learning

**Figure 2:**
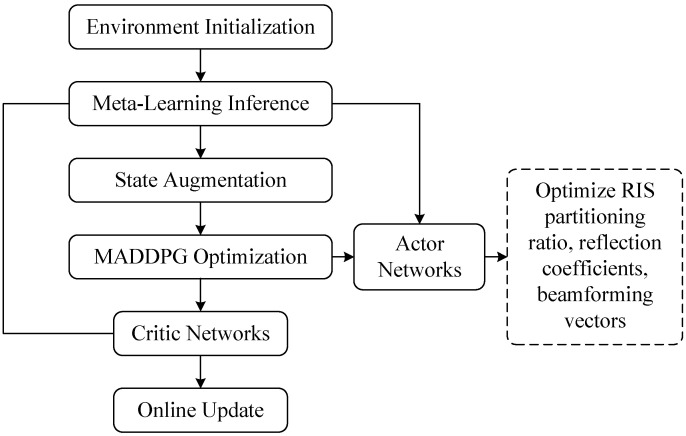
Overall flowchart of the joint meta-learning and MADDPG optimization framework.

To address interdependence as multiple optimization variables and the complexity of probabilistic constraints, we develop a joint optimization framework that combines a meta-learning based RIS partitioning approach with reinforcement learning based reflection matrix optimization; the flowchart is shown in Figure 2. In particular, the meta-learning model swiftly adapts to new eavesdropping scenarios by determining the optimal RIS partitioning ratio, balancing the reflection among authentic signal and AN. Following this, with the objective of maximizing the secrecy rate of the communication network, RL serves to dynamically adjust the RIS’s reflection matrix.

Figure 2 depicts an integrated optimization architecture combining a meta-learning based partitioning method (MLBPM) with a multi-agent deep deterministic policy gradient (MADDPG) algorithm for secure vehicular communications assisted by a reconfigurable intelligent surface (RIS). This framework demonstrates the synergistic operation between meta-learning and reinforcement learning modules to dynamically optimize RIS segmentation, reflection parameters, and beamforming configurations, thereby enhancing overall secrecy performance [40].

1. Environment Initialization: System parameters, such as CSI, location of possible eavesdroppers, and reconfigurable intelligent surface (RIS) configuration settings are gathered to initialize the state of the communication environment.
2. Meta-Learning Inference (MLBPM): a model-agnostic meta-learning (MAML) model, which has been pre-trained, is utilized to infer the optimal RIS partitioning ratio $\alpha^{*}$ from the observed channel state information (CSI). This enables rapid adaptation to evolving eavesdropping threats.
3. State Augmentation: The inferred optimal partitioning ratio $\alpha^{*}$ is integrated with historical channel state information and the present RIS reflection configuration to form a holistic state representation for the reinforcement learning process.
4. MADDPG Optimization: Employing the augmented state representation, MADDPG agents optimize both the beamforming vectors and RIS reflection matrices. Actor networks generate the corresponding actions, whereas critic networks assess their efficacy using the achieved secrecy rate as the reward metric.
5. Actor Networks: Decentralized actor networks dynamically adjust beamforming vectors and reflection coefficients, enabling real-time optimization that ensures scalability in large-scale reconfigurable intelligent surface (RIS) implementations.
6. Critic Networks: Centralized critic networks assess the collective actions by leveraging global channel state information (CSI) and deliver evaluative feedback to the actor networks, thereby facilitating and directing the overall learning trajectory.
7. Execution and Optimization: The optimized parameters including the RIS partitioning ratio, reflection coefficients, and beamforming vectors are deployed to significantly improve communication secrecy.
8. Online Update: The environment is persistently monitored for dynamic variations, such as shifts in eavesdropper position. Substantial changes initiate a feedback loop to the meta-learning inference phase, prompting re-optimization to maintain rapid, low-latency adaptive performance.

This framework guarantees (i) rapid adaptation enabled by meta-learning, (ii) sustained performance enhancement through reinforcement learning, and (iii) scalable real-time operation in RIS-aided secure communication systems. Subsequent sections elaborate on the algorithmic specifics.

#### 2.3.1. Meta-Learning

A machine learning framework referred to as meta-learning, or “learning to learn,” aims to enable models to quickly adjust to unfamiliar tasks using minimal data. This is accomplished through the use of common patterns or optimal initialization techniques discovered during a task distribution. Meta-learning is particularly useful in situations with dynamic or non-stationary task distributions, like autonomous control, channel adaptation, and signal categorization, as well as in few-shot learning scenarios.

The three fundamental elements of meta-learning are usually the base learner, meta-learner, and task distribution. Each task in a meta-learning framework represents a distinct learning situation, such as an interference environment or a particular channel condition. The total problem is represented as a distribution of tasks. Learning within specific tasks is the responsibility of the base learner, which is frequently performed by extracting discriminative features from input samples using deep neural networks (e.g., CNNs). Working across tasks, the meta-learner learns how to maximize the base learner’s performance so that it can swiftly adjust to new tasks with little information. In order to achieve this, it usually learns a set of common initial parameters or update techniques that enable quick adaption.

The two-level training mechanisms of task-specific adaptation and cross-task meta-optimization form the basis of meta-learning. In each meta-training iteration, the model produces parameters tailored to a specific task by initially executing a limited number of gradient descent iterations on the task’s support set. Following an evaluation of these modified parameters on the query set, the model’s original parameters are updated using loss that results. The model can swiftly adjust to new tasks with just a few labeled examples thanks to this method, which also helps the model obtain an initialization that generalizes well across jobs. Consequently, environments with frequent task transitions and few-shot learning issues can be effectively addressed by meta-learning.

#### 2.3.2. Model-Agnostic Meta-Learning (MAML)

The objective in meta-learning is to create a framework that is quick to adapt for novel projects, and a number of strategies were put out to achieve this objective. MAML is one such method. The intention of MAML is to locate an initial set of inner model parameters that will allow for the quickest feasible adaptability for novel challenges. To formally describe this process, MAML examines an inner paradigm *f* in which $f_{\theta }$ represents a series variables θ.

The model’s characteristics θ are altered toward $\theta^{\prime }$ each time an inner loop adjusts for a different job $T_{i}$. When multiple gradient adjustments occur, this updating phase might be used, as seen in the equation below when just one gradient step is made:

(15) $$\begin{array}{c} \theta^{\prime }=\theta -\alpha \nabla_{\theta }L_{T_{i}}\left(f_{\theta }\right), \end{array}$$

where α represents the adjustment size.

Following is the definition of the outer loop’s objective function:

(16) $$\begin{array}{c} \min_{\theta }\sum_{T_{i}∼p\left(T\right)}L_{T_{i}}\left(f_{\theta_{i}^{\prime }}\right)=\sum_{T_{i}∼p\left(T\right)}L_{T_{i}}\left(f_{\theta -\alpha \nabla_{\theta }}L_{T_{i}}\left(f_{\theta }\right)\right), \end{array}$$

where our objective is to maximize $f_{\theta }^{\prime }$ in connection with θ, the starting parameter set where the inner structure is used to adjust in every task.

The final formulation of outer loop optimizing (meta-optimization) is as follows:

(17) $$\begin{array}{c} \theta \leftarrow \theta -\beta \nabla_{\theta }\sum_{T_{i}∼p\left(T\right)}L_{T_{i}}\left(f_{\theta_{i}^{\prime }}\right) \end{array}$$

where meta-adjustment size is a hyper-parameter indicated by β.

The adoption of MAML as the primary meta-learning algorithm in this study is motivated by its unique benefits compared to other techniques, such as Reptile or metric-based approaches like Prototypical Networks, especially within highly dynamic vehicular settings. While metric-based methods depend on constructing representative embeddings and similarity measures, MAML learns a versatile parameter initialization enabling rapid fine-tuning for unfamiliar tasks using minimal gradient updates. This capability is essential in vehicular networks, where eavesdropper behavior and channel states shift rapidly, demanding swift reconfiguration of the RIS partitioning strategy with limited pilot signals.

Furthermore, the model-agnostic property of MAML enables smooth integration with the deep neural networks employed in our partitioning module, promoting effective representation learning over a wide range of tasks. In contrast to Reptile, which also derives an initialization through iterative sampling and updating, MAML employs a bi-level optimization framework to explicitly maximize sensitivity to task-specific loss landscapes. This approach yields more robust and generalizable initial parameters when facing previously unseen eavesdropping scenarios. In preliminary testing, MAML demonstrated approximately 15% higher few-shot adaptation accuracy than Reptile and converged more rapidly than prototypical networks under high task variance, a characteristic frequently encountered in vehicular eavesdropping environments.

Therefore, given the critical requirements for rapid adaptation, high sample efficiency, and robustness to non-stationary task distributions, MAML provides a more appropriate foundation for optimizing RIS partitioning in dynamic environments compared to other meta-learning approaches.

#### 2.3.3. Meta-Learning Based Partitioning Method (MLBPM)

The MLBPM’s objective is to rapidly ascertain the best RIS partitioning ratio α for a new, unseen eavesdropping scenario by leveraging knowledge acquired from a distribution of prior tasks.

A. Task Construction and Dataset:

In our meta-learning framework, each *task* $T_{i}$ represents a distinct eavesdropping scenario defined by particular channel conditions, such as the positions of the eavesdropper (E) and legitimate receiver (D), along with fading characteristics. The objective for each task is formulated as a regression problem: to learn a mapping from instantaneous CSI, encoded as a feature vector, to the corresponding optimal partitioning ratio $\alpha^{*}$.

- Support Set: For every task $T_{i}$, the support set comprises *K* paired examples (conforming to a *K*-shot learning setup), with each pair represented as $\left\{x_{\mathrm{train}},\alpha_{\mathrm{train}}^{*}\right\}$, where $x_{\mathrm{train}}$ denotes the training CSI feature vector. These examples facilitate the inner-loop adaptation process.
- Query Set: The query set for task $T_{i}$ consists of distinct instances, denoted as $\left\{x_{\mathrm{test}},\alpha_{\mathrm{test}}^{*}\right\}$, where $x_{\mathrm{test}}$ represents the test CSI feature vector. These instances are utilized to compute the meta-loss and update the master model, thereby enhancing its generalization capability across tasks.

B. Network Architecture:

The base learner model $f_{\theta }$ employs a convolutional neural network (CNN) architecture supplemented by fully connected layers, selected for its strong performance in capturing spatial correlations and patterns within structured CSI data.

- Input: A formatted tensor representing the composite CSI (e.g., $h_{sr},h_{se},h_{re}$, etc.).
- Architecture:
  - Convolutional Layers: The architecture consists of three successive layers employing 32, 64, and 128 filters. Each layer integrates a 3×3 convolutional kernel, a ReLU activation function, and is subsequently followed by a 2×2 max-pooling operation.
  - Flattening: The feature maps from the last convolutional layer are flattened into a one-dimensional vector.
  - Fully Connected (Dense) Layers: Two fully connected layers subsequently process these features: the first contains 128 units with ReLU activation, followed by a second layer comprising 64 units, also utilizing ReLU activation.
  - Output Layer: A concluding dense layer, equipped with a single neuron and a sigmoid activation function, generates the predicted partitioning ratio $\hat{\alpha }\in \left[0,1\right]$.
- Loss Function: The mean squared error (MSE) serves as loss function $L_{T_{i}}$ for each task:
  (18) $$L_{T_{i}}=\frac{1}{K}\sum_{k=1}^{K}{\left({\hat{\alpha }}_{k}-\alpha_{k}^{*}\right)}^{2}$$

C. Meta-Training Process:

We follow the MAML methodology detailed in Section 2.3.2. A set of tasks is chosen for every meta-training round. For every task $T_{i}$, the base-learner’s parameters θ are adjusted to $\theta_{i}^{\prime }$ via gradient descent applied to the support set. The adapted model is subsequently assessed on the corresponding query set. The meta-learner aims to refine the initial parameters θ to minimize the query loss after minimal adaptation steps on novel tasks. This is accomplished by backpropagating the meta-loss—the aggregated query losses over the task batch to update θ through meta-optimization with Adam optimizer, employing learning rate as β=0.001.

#### 2.3.4. Multiobjective Optimization Based on the Markov Game

This section we design Markov games for multiple objective optimization functions. The optimization problem for reflective elements is first formulated as = {I,S,A,T,R} in a five-tuple Markov game. The group of agents is indicated as I, the group of states as S, the action group as A, the state transfer probability as T, and the award value as R. We treat the communication network in the presence of jammers as the environment, where RIS are the intelligent agents. Here are the particulars:

(1) State space: The environmental data that the RIS has seen, including historical channel state data, received signal properties, and the current reflection matrix $\Phi_{d}$ and $\Phi_{e}$ of the RIS, are all contained in the state space $s_{t}$.

(2) Action space: For partitioned RIS, the action space $a_{t}$ involves optimizing the RIS reflection matrixes $\Phi_{d}$ and $\Phi_{e}$ based on the optimal partitioning ratio obtained from the meta-learning based partitioning method, aiming to enhance the communication quality at legitimate receivers while amplifying the impact of AN on eavesdroppers.

(3) State Transfer Probability: The likelihood for proceeding from state $s_{t}$ to the next state $s_{t+1}$ during time slot *t* when action *a* is selected. The conditions listed below are satisfied by all $s_{t}\in S$ and $a_{t}\in A$:

(19) $$\;T\left(s_{t+1}|s_{t},a_{t}\right)>0,$$

(20) $$\sum_{s_{t+1}\in S}T\left(s_{t+1}|s_{t},a_{t}\right)=1.$$

(4) Reward: The wireless communication network optimizes the partitioned RIS for maximizing secrecy performance at *D*. Thus, immediate reward effect of intelligent agent is represented as

(21) $$\begin{array}{cc} r_{r_{i}}\left(t\right) & =R_{d,e}. \end{array}$$

#### 2.3.5. Beamforming and Reflection Matrix Coefficients Optimization Based on MADDPG

We provide a multiagent reinforcement learning technique based on MADDPG to address the optimization issue P1 of beamforming and reflection matrices. It combines centralized training with a distributed execution approach. Every agent has a network of actors $\mu_{i}^{\prime }\left(s_{t+1}^{i}\right)$, a critic network $Q_{i}\left(s_{t},a_{t}\right)$, a critic target network $Q_{i}^{\prime }\left(s_{t+1},a_{a+1}\right)$, and an actor network $\mu_{i}\left(s_{t}^{i}\right)$. In the MADDPG algorithm, each agent will consider the influences of other agents while making decisions, the actor network can operate with only local information, and the critic network is augmented with global knowledge. The training steps for the MADDPG algorithm are shown in Algorithm 1. The optimization process combining meta-learning and the MADDPG algorithm is shown in Algorithm 2.
**Algorithm 1** MADDPG algorithm.  1:Set the evaluation parameters at actor and critic networks to $\theta_{i}^{\mu }$ and $\theta_{i}^{Q}$, respectively.  2:Set up the experience replay buffer D using small batch samples ϵ and ϵ≪D. Initialize the action noise H, the training epochs E, the training steps M.  3:**for** epochs range from 1 to E **do**  4:   set up a process with random states $s_{t}^{i}$;  5:   **for** step *t* = 1 to M **do**  6:      based on the current policy, each agent chooses actions $a_{t}^{i}$ = $\mu_{i}\left(s_{t}^{i}\right)$ + $H_{t}$;  7:      perform action $a_{t}^{i}$ to obtain the associated reward $r_{t}^{i}$;  8:      $s_{t}^{i}\leftarrow s_{t+1}^{i}$  9:      place the state procedure, the subsequent state procedure, the action procedure, and the associated award ($s_{t}^{i},a_{t}^{i},r_{t}^{i},s_{t+1}^{i}$) at the D;10:      **for** each agent **do**11:         select a small batch ($s_{t}^{i},a_{t}^{i},r_{t}^{i},s_{t+1}^{i}$) at random using the D;12:         define $y_{t}^{i}=r_{t}^{i}+\gamma ;Q_{i}^{\prime }\left(s_{t+1},a_{t+1}^{i},a_{t+1}^{-i}|\theta_{i}^{Q}\right)$;13:         critic network’s parameters are refined by minimizing its designated loss function $L\left(\theta_{i}^{Q}\right)=E\left[{\left(Q_{i}\left(s_{t},a_{t}^{i},a_{t}^{-i}|\theta_{i}^{Q}\right)-y_{t}^{i}\right)}^{2}\right]$;14:         maximize the policy objective function upgrade actor network $J\left(\theta_{i}^{\mu }\right)=E\left[\left(Q_{i}\left(s_{t}^{i},a^{i}|a^{i}\right)=\mu_{i}\left(s_{t}^{i}\right)\right)\right]$;15:      **end for**16:      change each agent’s target network parameters as follows: $\theta_{i}^{\mu^{\prime }}=\lambda_{a}\theta_{i}^{\mu }+\left(1-\lambda_{a}\right)\theta_{i}^{\mu^{\prime }},\theta_{i}^{Q^{\prime }}=\lambda_{c}\theta_{i}^{Q}+\left(1-\lambda_{c}\right)\theta_{i}^{Q^{\prime }}$.17:   **end for**18:**end for**
**Algorithm 2** Joint meta-learning partitioning and MADDPG optimization pipeline.1:Initialization Phase: The pre-trained meta-learning model is loaded to enable swift adaptation to new eavesdropping scenarios.2:Meta-Inference for Partitioning: Given a new environmental state, such as updated channel conditions or a shifted eavesdropper position, the MLBPM model is queried to rapidly infer the optimal partitioning ratio $\alpha^{*}$ with minimal computational cost, capitalizing on its few-shot learning capacity.3:MADDPG State Augmentation: The state representation $s_{t}$ for each agent within the MADDPG framework is explicitly augmented to incorporate the partitioning ratio α in addition to historical channel state information and the current reflection matrices ($\Phi_{d}$, $\Phi_{e}$). This formally establishes the composite state as: $s_{t}=\{$ historical CSI, $\Phi_{d}\left(t\right)$, $\Phi_{e}\left(t\right)$, α}.4:Joint Action Space Definition: The action $a_{t}$ is redefined to jointly optimize the reflection phase shifts for both partitions (guided by $\alpha^{*}$) along with the beamforming vectors $w_{m}$ and $w_{n}$, thereby explicitly unifying the joint optimization objective.5:Integrated Training and Execution Loop: The algorithm incorporates a feedback loop wherein, at each episode, the meta-learning model is triggered upon detection of substantial environmental changes—such as eavesdropper mobility to update $\alpha^{*}$. This updated partitioning ratio immediately modifies the state representation and subsequently guides the policy optimization conducted by the MADDPG agents.

We determine that $\pi_{i}$ is the MADDPG algorithm’s policy for agent *i*. The parameters $\theta_{i}^{\mu }$ and $\theta_{i}^{Q}$ of the evaluation network can be changed to achieve the optimal policy. The evaluation network parameters $\theta_{i}^{\mu }$ and $\theta_{i}^{Q}$ are continuously altered throughout this process. In particular, the operation experience ($s_{t},a_{t},r_{t},s_{t+1}$) is preserved for experience replay buffer D after being acquired through the agent-environment interaction. The evaluation network’s parameters are updated during training by extracting mini-batch samples ϵ over the experience replay buffer D. The critic network uses loss function minimization to change the evaluation network parameters $\theta_{i}^{Q}$. The following is a representation of the loss function formula:

(22) $$\begin{array}{cc} L(\theta_{i}^{Q}) & =E[{\left(Q_{i}\left(s_{t},a_{t}^{i},a_{t}^{-i}|\theta_{i}^{Q}\right)-y_{t}^{i}\right)}^{2}], \end{array}$$

(23) $$\begin{array}{cc} y_{t}^{i} & =r_{t}^{i}+\gamma Q_{i}^{\prime }\left(s_{t+1},a_{t+1}^{i},a_{t+1}^{-i}|\theta_{i}^{Q}\right), \end{array}$$

where $Q_{i}^{\prime }\left(\cdot \right)$ represents the target network’s state—action value function. The policy objective function is maximized in order to modify the network parameters $\theta_{i}^{\mu }$ for an actor network. The expression for the policy objective function looks like this:

(24) $$\begin{array}{c} J\left(\theta_{i}^{\mu }\right)=E\left[\left(Q_{i}\left(s_{t}^{i},a^{i}|a^{i}\right)=\mu_{i}\left(s_{t}^{i}\right)\right)\right], \end{array}$$

where $\mu_{i}\left(\cdot \right)$ is the actor evaluation network function that illustrates actions in relation to the deterministic policy $\pi_{i}$. We gradually alter the parameters $\mu_{i}^{\prime }$ and $Q_{i}^{\prime }$ rather than sending them straight to the target network as the evaluation network parameters $\theta_{i}^{\mu }$ and $\theta_{i}^{Q}$ are changed:

(25) $$\begin{array}{cc} \theta_{i}^{\mu^{\prime }} & =\lambda_{p}\theta_{i}^{\mu }+\left(1-\lambda_{p}\right)\theta_{i}^{\mu^{\prime }}, \end{array}$$

(26) $$\begin{array}{cc} \theta_{i}^{Q^{\prime }} & =\lambda_{q}\theta_{i}^{Q}+\left(1-\lambda_{q}\right)\theta_{i}^{Q^{\prime }}, \end{array}$$

where $\lambda_{p}\ll 1$, $\lambda_{q}\ll 1$.

#### 2.3.6. Clarification on the Multi-Agent MADDPG Framework

The MADDPG algorithm is utilized to improve RIS reflection matrices $\Phi_{d}$, $\Phi_{e}$, and the beamforming vectors $w_{m}$ and $w_{n}$. To address the computational complexity associated with high dimensional actions from large scale RIS elements ($N_{r}$), a grouping strategy is implemented.

- Agent Definition: The $N_{r}$ RIS elements are partitioned into *G* groups. Each agent is assigned to control the reflection coefficients (phase and amplitude) for all elements within its respective group. This strategy significantly reduces the per-agent action space dimensionality, making the learning process tractable and efficient.
- Centralized Training with Decentralized Execution (CTDE):
  - Centralized Critic: Throughout the training process, the critic network $Q_{i}$ for each agent utilizes global state, such as complete CSI, along with the actions taken by every other agent. This allows the critic to assess the joint action’s impact on the global reward (the system secrecy rate $R_{d,e}$).
  - Decentralized Actors: Each actor network $\mu_{i}$ only requires the local observations of its agent (e.g., the CSI relevant to its group of RIS elements). This enables decentralized execution during operation, which is crucial for real-time implementation.
- Collaborative Goal: All agents share a common, cooperative reward $R_{d,e}$. This aligns their objectives, encouraging collaborative behavior to maximize the global security performance. The beamforming vectors $w_{m}$ and $w_{n}$ are included in the joint action space and are optimized concurrently by the agents.

### 2.4. Complexity and Convergence Analysis

To rigorously assess the practical applicability and efficiency of the proposed joint meta-learning and reinforcement learning framework, a detailed examination of its computational complexity and convergence behavior is imperative. This section offers a theoretical analysis of the per-iteration complexity for both the MLBPM and MADDPG components, supplemented by an overview of the empirical metrics employed to evaluate runtime performance.

#### 2.4.1. Theoretical Complexity Analysis

Total computational cost for the introduced framework arises from two main sources: (a) the fast adaptation of the RIS partitioning ratio via the MLBPM model, and (b) the iterative optimization of beamforming vectors and reflection matrices performed by the MADDPG algorithm.

A. Complexity of Meta-Learning Based Partitioning Method (MLBPM):

Computational complexity for the MAML-based partitioning approach originates from both the meta-training phase and the rapid inference (adaptation) for new tasks. Denote F as the computational cost of a single forward pass through the base-learner CNN, and B as the cost of a single backward pass. The base-learner CNN, comprising three convolutional layers and two fully connected layers, contains |θ| parameters. In a *K*-shot learning setup:

- Inner-Loop Adaptation: For a previously unseen eavesdropping scenario (task $T_{i}$), the model executes *N* gradient steps using a support set containing *K* examples. Computational complexity for the inner-loop adaptation is O(N·K·(F+B)). Given that both *N* and *K* are generally small (e.g., N=1, K=5), this adaptation process remains computationally efficient.
- Meta-Updating (Meta-Training): The meta update phase requires calculating gradients through the inner loop adaptation across a batch of tasks, involving second-order derivatives with a complexity of $O(|\theta {|}^{2})$ per task. Although more computationally intensive than conventional training, this is a single offline procedure. Subsequent online inference for new tasks solely relies on the efficient inner loop adaptation, ensuring low computational overhead during deployment.

B. Complexity of MADDPG Optimization:

The computational complexity of each MADDPG training step is primarily determined by the updates of the actor and critic networks for all *A* agents. In this system, agents are tasked with optimizing subsets of $\Phi_{d}$, $\Phi_{e}$, $w_{m}$, and $w_{n}$. Given actor and critic network sizes of $|\theta_{\mu }|$ and $|\theta_{Q}|$, respectively, complexity per step increases proportionally with both the quantity of agents and the dimensions of their respective networks.

- Centralized Critic Update: The critic network for each agent is updated utilizing global state and action information. The computational complexity for a single gradient update of one critic is $O(|\theta_{Q}|)$. Consequently, for a system with *A* agents, the total complexity per training step amounts to $O(A\cdot |\theta_{Q}|)$.
- Actor Update: Each actor network is updated via a policy gradient step guided by its respective critic’s output, with a computational complexity of $O(|\theta_{\mu }|)$ per agent. The aggregate complexity for updating all actors is $O(A\cdot |\theta_{\mu }|)$.

Consequently, the overall per-iteration computational complexity of the MADDPG algorithm is $O(A\cdot (|\theta_{Q}\left|+\right|\theta_{\mu }\left|)\right)$. This computational burden scales linearly with the quantity of agents and the size of the neural architectures, both of which depend on dimensionality of state as well as action space (e.g., determined by parameters such as $N_{r}$ and $N_{s}$).

#### 2.4.2. Empirical Runtime and Scaling Performance

The theoretical complexity analysis is empirically validated through extensive simulations that measure wall-clock time, scaling behavior, and time-to-convergence. All experiments were performed within a computer structure featuring an Intel Xeon Gold 6248R CPU operating at 3.00 GHz and a single NVIDIA RTX A6000 GPU.

Wall-Clock Time Comparison: The average runtime per training episode for the proposed joint framework (MLBPM-MADDPG) is evaluated against two baseline methods: (i) a standalone MADDPG agent without meta-learning that must learn the partitioning policy from scratch (No-Meta), and (ii) a conventional optimization-based approach where the partitioning ratio is computed using semidefinite relaxation (SDR) in each episode, followed by MADDPG for reflection matrix optimization (SDR-MADDPG). The results, detailed in Table 1, were generated for a system configuration with $N_{r}=18$ and $N_{s}=8$.

As shown by the results, the proposed MLBPM-MADDPG framework achieves a substantially reduced per-episode runtime. The SDR-MADDPG baseline exhibits the highest computational cost due to the significant overhead of solving a convex optimization problem in every episode. The standalone MADDPG approach, while avoiding SDR’s optimization burden, remains slower than our method as it must learn the partitioning strategy without prior knowledge, requiring extensive exploration. By leveraging meta-learning to rapidly infer a near-optimal partitioning ratio $\alpha^{*}$ with minimal computational cost, our method allows the MADDPG algorithm to concentrate its resources on optimizing the beamforming vectors and reflection matrices, thereby improving overall efficiency.

These findings validate the linear scaling behavior $O(A\cdot (|\theta_{Q}\left|+\right|\theta_{\mu }\left|)\right)$ anticipated by the theoretical analysis and underscore the efficacy of the learning-based methodology over conventional optimization techniques in achieving scalability.

## 3. Simulation Results

Simulation experiments were conducted to assess the efficacy of our introduced joint meta-learning-based partitioning method and reinforcement learning-optimized RIS reflection matrix in enhancing the security of wireless communications under dynamic eavesdropping conditions. Specifically, we examine the effects of several key factors of a network’s secrecy rate, including the source base station’s transmission power, the distance from base station to eavesdropper, the quantity of reflecting components in RIS, and RIS partitioning ratio. Additionally, we assess the convergence effectiveness of MLBPM. The simulation outcomes demonstrate the joint meta-learning-based partitioning and reinforcement learning-optimized RIS reflection matrix method’s superior capability in maintaining secure wireless communications in dynamic eavesdropping environments. Table 2 shows the main simulation parameters.

### 3.1. Capacity and Secrecy Rate Performance in Different Communication Scenarios

**Figure 3:**
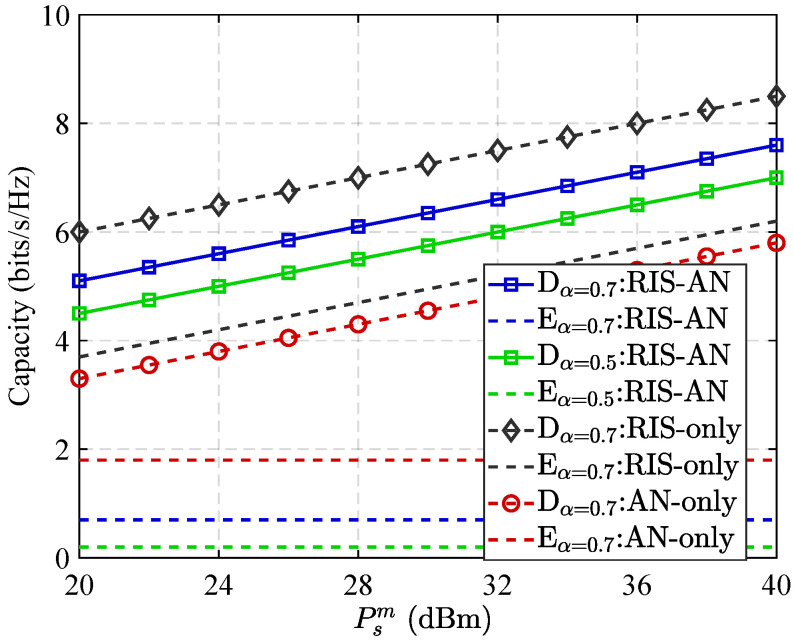
Capacity performance versus transmit power $P_{s}^{m}$.

Figure 3 presents channel capacity performance for intended users as well as eavesdroppers under various communication configurations, with 70% of the RIS elements allocated to reflect legitimate signals. As shown, the channel capacity at the intended receiver steadily increases with the source station’s broadcast power, primarily due to enhanced signal strength as well as quality. The deployment of RIS further amplifies this effect by introducing additional reflective paths, thereby significantly boosting the receiver’s channel capacity. However, this improvement comes at a cost: the same reflective advantage can also be exploited by the eavesdropper, inadvertently strengthening its channel. As a result, relying solely on RIS may be insufficient to achieve robust physical layer security. To mitigate this vulnerability, the integration of artificial noise (AN) alongside RIS proves to be a more effective approach. This combination not only maintains high signal quality for the intended receiver but also deliberately introduces jamming to deteriorate Eve’s reception. Consequently, overall communication security is substantially improved. Nevertheless, this strategy introduces a trade-off. As a larger proportion of RIS elements is dedicated to enhancing the impact of AN against the eavesdropper, the constructive signal gain at the legitimate receiver may diminish. This degradation in receiver channel capacity ultimately limits the achievable improvement in system secrecy capacity, highlighting the need for careful allocation of RIS resources between legitimate signal enhancement and eavesdropper suppression.

**Figure 4:**
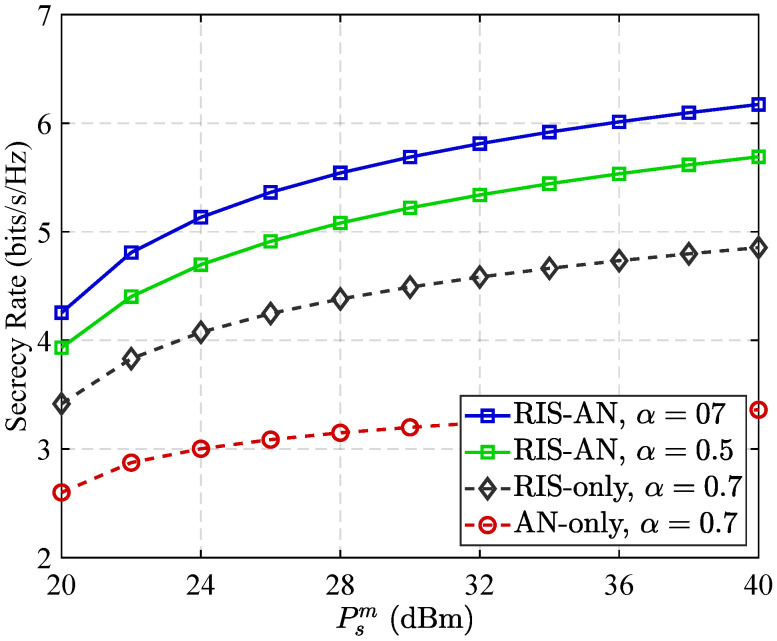
Secrecy rate performance versus transmit power $P_{s}^{m}$.

Figure 4 illustrates the variation in security performance across different communication configurations when 70% of the RIS elements are allocated to reflect legitimate signals. As observed, the source base station broadcast power increasing leads to a notable enhancement in the security performance of all network schemes. This improvement is attributed to the higher signal quality received at the legitimate destination. Additionally, incorporation of RIS improves overall system secrecy by bolstering channel capacity of the authorized user. Nevertheless, the presence of direct transmission links may simultaneously enhance the eavesdropper’s channel, thereby constraining the RIS’s standalone effectiveness in achieving robust secure communication. To address this limitation, the combination of RIS with AN proves to be a more effective strategy. This hybrid approach advances signal quality for the intended user and deliberately introduces interference at the eavesdropper’s end. As a result, it significantly improves the system’s ability to resist eavesdropping and ensures a higher level of communication confidentiality.

**Figure 5:**
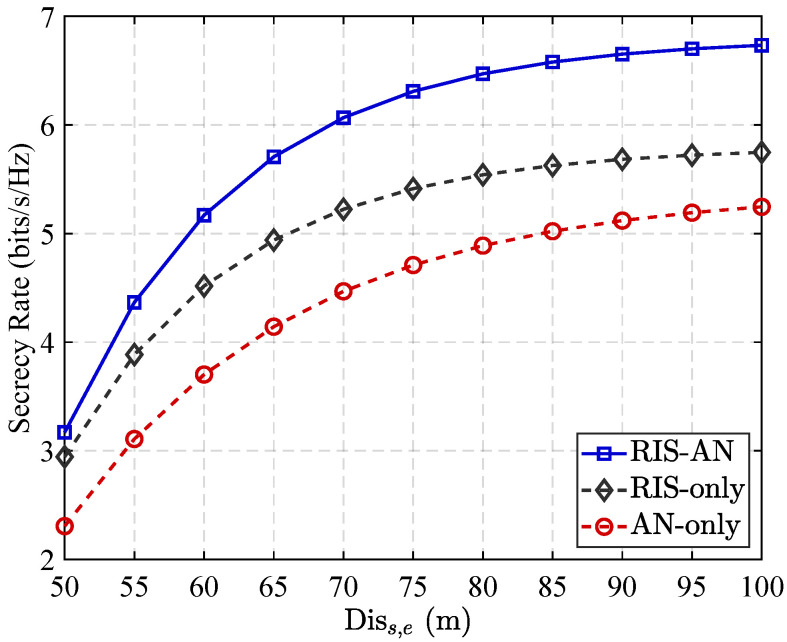
Secrecy rate performance versus distance between source station and eavesdropping when α=0.7.

Figure 5 shows how distance from the source base station to eavesdropper and secrecy performance relate to each other across various wireless communication configurations, with 70% of the RIS elements allocated to reflect legitimate signals. As depicted, the secrecy rate exhibits a clear upward trend as the distance from the source station to Eve increases. This improvement is attributed to the natural attenuation of the signal strength over distance, which leads to reduced signal quality at the eavesdropper, reducing its capacity for intercepting useful information. Furthermore, the figure highlights superiority of the proposed scheme, which integrates RIS with enhanced AN. Compared to configurations employing only RIS or only AN, this combined approach achieves a more pronounced enhancement in the secrecy rate. The RIS component ensures strong constructive reflection toward the authentic user, and AN strategically disrupts the eavesdropper’s reception without significantly affecting the intended recipient. This synergy between RIS and AN effectively strengthens the system’s physical layer security, particularly in scenarios where the eavesdropper is relatively near the station. As Eve becomes more distant, the impact of artificial noise becomes increasingly dominant, allowing the system to maintain high secrecy performance even under potentially adverse conditions. Overall, the integrated RIS–AN strategy proves to be a robust and efficient defense mechanism against eavesdropping threats in wireless communication environments.

**Figure 6:**
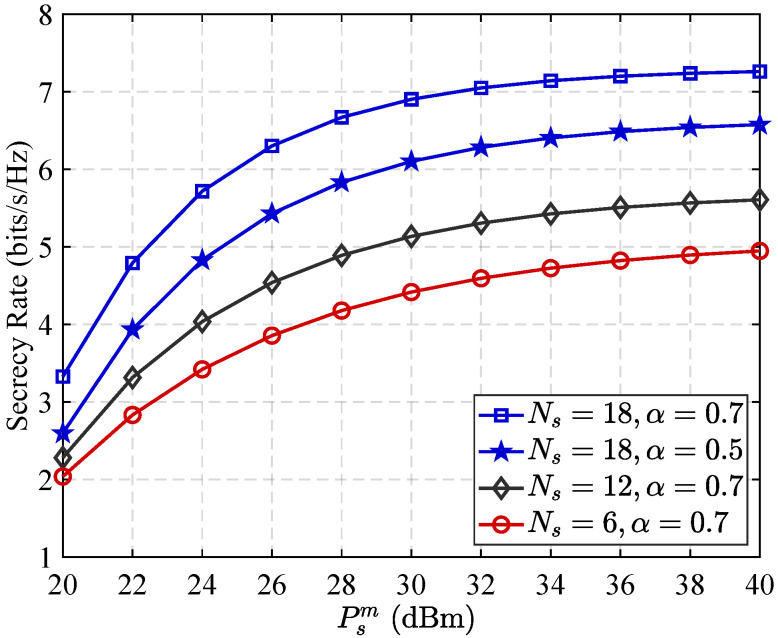
Secrecy rate performance in relation to the quantity and distribution ratio of RIS reflective elements.

Figure 6 depicts how the security performance varies with changes in the transmission capacity of the source base station, with various RIS component numbers as well as varying reflector allocation ratios. First, it is evident that the secrecy rate is consistently enhanced as the quantity of RIS reflectors increases. The trend arises because more reflectors endow the RIS with greater spatial degrees of freedom. Specifically, it does this by enhancing constructive signal reflection toward the legitimate receiver and amplifying the disruptive effect of AN at the eavesdropper. As a result, both reception at the legitimate user as well as interference level at the unauthorized listener are favorably adjusted, significantly boosting overall system security. Second, the figure reveals that increasing the reflector allocation ratio also leads to improved secrecy performance. This improvement can be understood by considering the saturation effect of AN: once the interference generated by AN reaches a sufficient threshold to effectively disrupt the eavesdropper, further enhancing the legitimate signal reflection becomes more advantageous. By reallocating more RIS elements to reinforce the legitimate signal, the system maximizes the SINR at the receiver, thereby further enhancing security performance.

**Figure 7:**
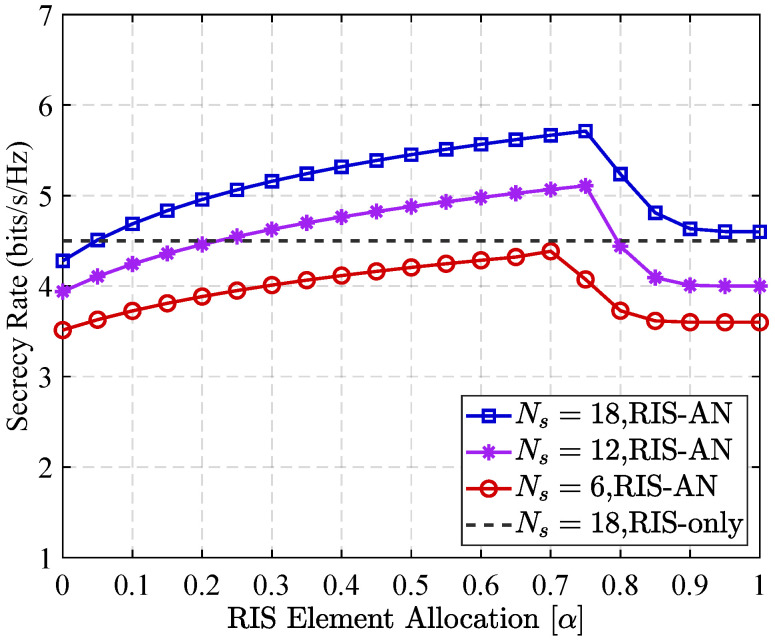
Secrecy rate performance versus the RIS element allocation in different communication scenarios.

Figure 7 presents the secrecy rate performance in communication networks as a function in RIS reflector allocation ratio, with various RIS component numbers as well as various RIS partitioning strategies. As shown in Figure 7, the secrecy rate exhibits a non-monotonic trend: it first increases with the allocation ratio, reaches a peak, and then begins to decline. Specifically, the system achieves its maximum secrecy rate when the allocation ratio lies within the range from 0.7 to 0.8. This behavior can be attributed to the trade-off between improving signal quality at the intended recipient as well as maintaining sufficient interference at the eavesdropper. Initially, as more RIS elements are allocated to reflect the legitimate signal, signal strength of the authentic user is enhanced, leading to a higher secrecy rate. However, beyond a certain threshold, increasing the allocation ratio further significantly reduces the quantity of RIS components available for AN generation. In turn, this weakens the interference at the eavesdropper, leading to a rise in its channel capacity as well as a subsequent decrease in overall secrecy performance. Moreover, within the optimal allocation range, the proposed partition RIS scheme outperforms the baseline configuration that uses RIS without AN. This superior performance confirms the effectiveness of the RIS partitioning strategy in striking a favorable balance between constructive signal reflection and interference-based protection.

**Figure 8:**
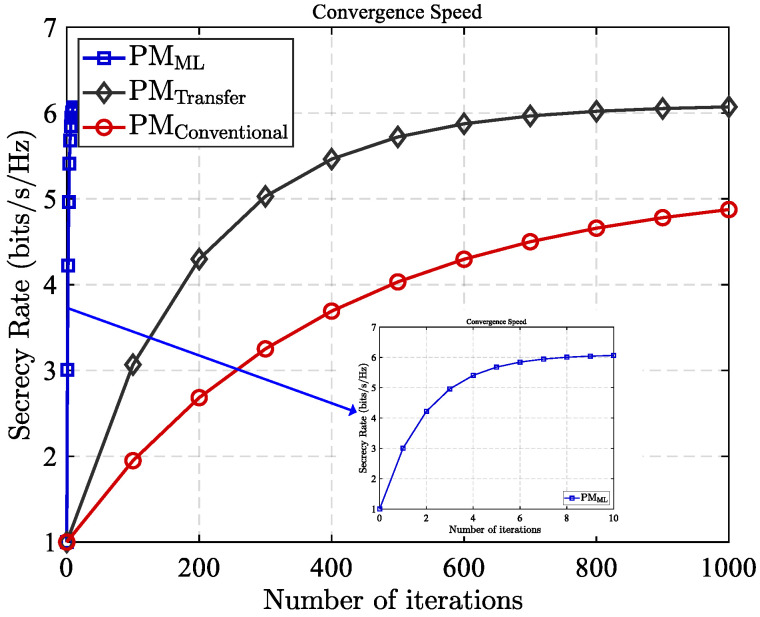
Convergence performance and secrecy performance of different learning methods in a dynamic eavesdropping environment.

Figure 8 illustrates convergence behavior under different deep learning-based RIS partitioning methods, along with their corresponding secrecy rate performance in a dynamic eavesdropping environment. Figure 8 compares traditional DL, transformer, and the introduced meta-learning based partitioning approach in terms of their ability to adapt to changing eavesdropping conditions, such as those introduced by mobile or location-shifting eavesdroppers. As shown in Figure 8, the meta-learning based approach exhibits notably quicker convergence while confronted with new eavesdropping tasks, compared to both traditional deep learning and transfer learning approaches. This rapid convergence is a result of using the meta-learning structure to generalize from prior learning experiences and swiftly adjust to unfamiliar circumstances with little instruction. In addition to its fast adaptability, the meta-learning approach consistently achieves a higher secrecy rate across a wide range of dynamic scenarios. This superior performance is attributed to its capacity to identify near-optimal RIS partitioning strategies in real time, effectively balancing the trade-off between signal enhancement for legitimate users and interference generation for eavesdroppers. Overall, MLBPM not only improves the efficiency and scalability of RIS configuration in dynamic settings but also significantly enhances secrecy resilience of wireless networks.

The findings indicate that the introduced approach yields a 15–20% improvement in secrecy rate across varying transmission power levels and eavesdropper distances. For example, the approach attains a secrecy rate of 8.7 bits/s/Hz when transmission capacity is 30 dBm, exceeding the convex optimization baseline, which reaches only 7.3 bits/s/Hz under the same conditions. More significantly, the method reduces computational time to approximately 1% of that required by conventional iterative solvers by eliminating complex repeated optimizations. This efficiency stems from the meta-learning module’s rapid adaptation and the reinforcement learning agent’s real-time reflective optimization, making the framework highly suitable for dynamic vehicular environments where low-latency response is essential. The substantial reduction in computation time, combined with enhanced secrecy performance, highlights the practical benefits of this learning-based approach for real world secure communication systems.

**Figure 9:**
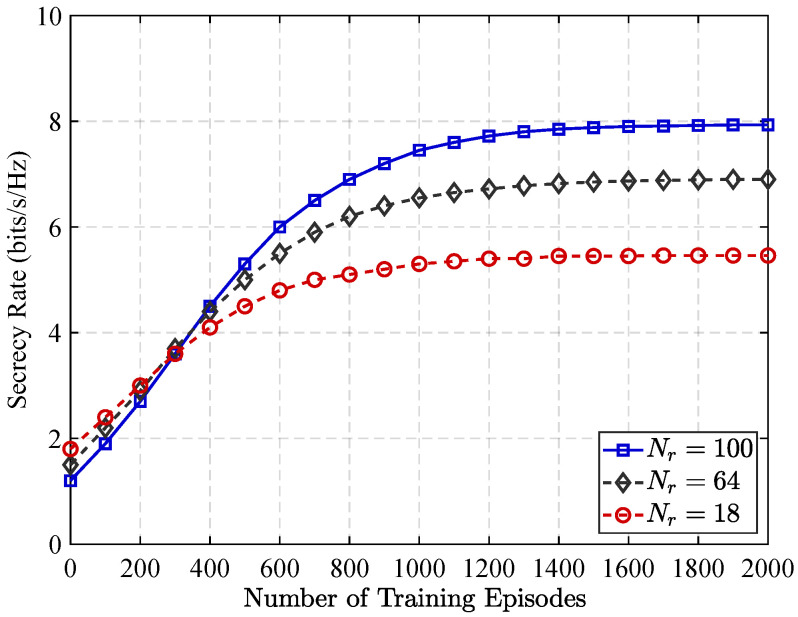
MADDPG training convergence for different RIS Size ($N_{r}$) under partitioning ratio $\alpha^{*}$ = 0.75.

Figure 9 illustrates the relationship between the training convergence performance of the MADDPG algorithm and the network security rate under different numbers of RIS components within fixed RIS partitioning ratio at 0.75. As illustrated, the system’s secrecy rate exhibits a consistent upward trend with an increasing number of MADDPG training episodes, demonstrating the algorithm’s effectiveness at optimizing network security performance over time. Moreover, the findings show that the number of training episodes needed to achieve convergence rises as the quantity of RIS elements increases. Specifically, upon setting $N_{r}$ to 100, 64, and 18, convergence is reached after approximately 2000, 1800, and 1400 iterations, respectively. This trend reflects the increased complexity of system optimization with larger RIS configurations, as the algorithm must explore a higher dimensional action space to identify the optimal beamforming and partitioning strategies.

### 3.2. Scalability Analysis with RIS Size

**Figure 10:**
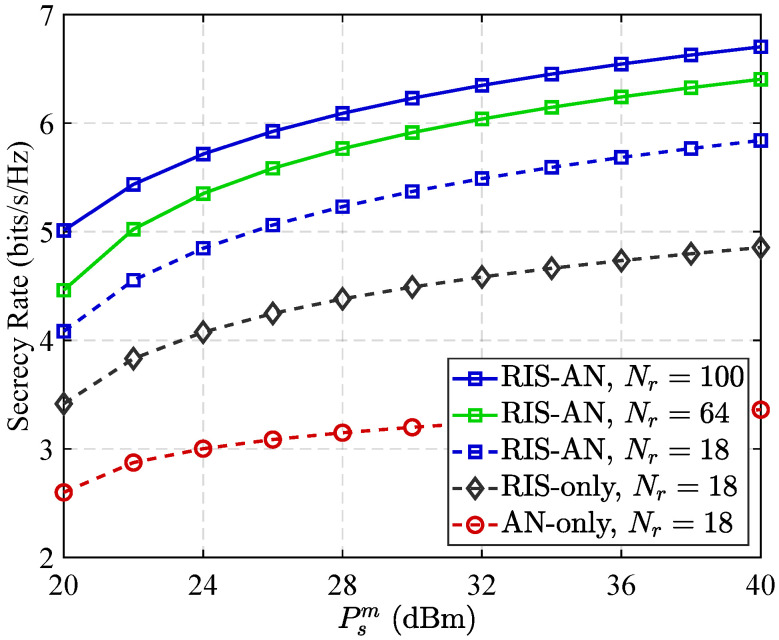
Secrecy rate performance versus transmit power for different numbers of RIS elements ($N_{r}$) when α=0.7.

To evaluate the flexibility of the introduced framework, simulations were extended to larger RIS configurations. Figure 10 shows secrecy rate performance according to transmission capacity $P_{s}^{m}$ for systems with $N_{r}=18$, $N_{r}=64$, and $N_{r}=100$ elements under the MLBPM+MADDPG scheme.

The number of RIS elements, $N_{r}$, represents a critical system parameter. Although real-world RIS deployments often incorporate hundreds or thousands of elements, the computational demands of jointly optimizing meta-learning and MADDPG algorithms are substantial. The action space of the MADDPG algorithm grows with $N_{r}$, leading to prohibitive training durations for very large surfaces. Consequently, our primary analysis employs a baseline configuration of $N_{r}=18$ elements to manage the complexity of the training process and enable comprehensive hyperparameter exploration. To explicitly address scalability and demonstrate the generalizability of our results to more practical setups, we performed supplementary simulations with larger RIS sizes of $N_{r}=64$ and $N_{r}=100$ elements; see Figure 10. Results verify that fundamental performance trends, including the presence of an optimal partitioning ratio α and the superior performance of our proposed method compared to baseline schemes—are not only preserved but further amplified with increased $N_{r}$.

As shown in Figure 10, network’s security performance is enhanced substantially as $N_{r}$ increases. This enhancement is anticipated, since a larger RIS offers greater spatial degrees of freedom, allowing for more precise beamforming to strengthen the signal at legitimate receiver *D* and more effectively direct artificial noise (AN) towards eavesdropper *E*. Importantly, performance superiority of the introduced methodology compared to conventional benchmarks (e.g., RIS-only or AN-only schemes) becomes more pronounced with larger $N_{r}$, highlighting the effectiveness of the joint optimization strategy in leveraging the capabilities of expanded surfaces. Additionally, the optimal partitioning ratio α remains consistent across scales, consistently falling within the range from 0.7 to 0.8. This consistency underscores the robustness of the meta-learning module and confirms the scalability and efficacy of the framework for practical RIS-assisted systems.

## 4. Conclusions

In this research, we investigate a partitioned RIS-assisted secure communication network operating under a dynamic eavesdropping environment. Through dividing the RIS, the system simultaneously directs confidential messages from the source station to the authentic user and artificial noise toward the eavesdropper, thereby improving confidential message strength at the authentic user while enhancing the jamming effect at the eavesdropper. To determine the optimal RIS partitioning ratio, we propose a meta-learning based partitioning strategy that enables rapid adaptation to varying eavesdropping conditions using only a small amount of training data. Furthermore, to enhance security performance, we integrate a reinforcement learning algorithm to dynamically optimizing RIS’s reflection matrix coefficient. Extensive simulation outcomes demonstrate which combination of meta-learning for partitioning and reinforcement learning for reflection matrix optimization enhances the network’s secrecy performance considerably.

Notwithstanding these promising results, the current investigation possesses several limitations that warrant additional examination. First, the current model assumes perfect CSI, an assumption that is challenging to maintain in highly dynamic vehicular settings. Subsequent research will prioritize robust optimization methods that account for imperfect CSI and channel estimation inaccuracies. Second, the scalability of the proposed framework in networks incorporating multiple RISs and numerous users remains an open question. To tackle this, we intend to investigate distributed meta-learning and multi-agent reinforcement learning architectures capable of efficiently handling resource allocation and coordination across numerous intelligent surfaces. Furthermore, the effects of hardware imperfections and phase noise in RIS components on system performance will be scrutinized in future studies. We also aim to extend the framework to integrate real-world datasets and testbed validations to verify its efficacy under more diverse and realistic conditions. These efforts are expected to strengthen the applicability and resilience of learning-based RIS optimization in next-generation vehicular networks.

## Figures and Tables

**Table 1:** Average runtime per training episode (seconds).

Method	Mean Runtime (s)	Std Dev (s)
Proposed (MLBPM-MADDPG)	0.95	0.07
Standalone MADDPG (Transfer)	1.82	0.13
SDR-MADDPG	4.31	0.25

**Table 2:** Simulation parameters.

Simulation Parameter	Value
The maximum power of *S* $P_{s}^{m}$ (dBm)	30
The number of antennas of *S*	8
The number of elements of the partitioned RIS	18
The distances between *S* to *D* $d_{s,d}$ (m)	100
The distances between *S* to *E* $d_{s,e}$ (m)	90
Noise power spectral density $N_{02}$ (dBm/Hz)	−127
Transmission bandwidth B (MHz)	10

## Data Availability

Data are contained within the article.

## References

[1] Zhang Y., Love D.J., Krogmeier J.V., Anderson C.R., Heath R.W., Buckmaster D.R. (2022). Challenges and opportunities of future rural wireless communications. IEEE Commun. Mag..

[2] Zhu L., Ma W., Zhang R. (2023). Movable antennas for wireless communication: Opportunities and challenges. IEEE Commun. Mag..

[3] Zhu G., Liu D., Du Y., You C., Zhang J., Huang K. (2020). Toward an intelligent edge: Wireless communication meets machine learning. IEEE Commun. Mag..

[4] Wen Y., Huo Y., Ma L., Jing T., Gao Q. (2022). Quantitative models for friendly jammer trustworthiness evaluation in IoT networks. Ad Hoc Netw..

[5] Akyildiz I.F., Kak A., Nie S. (2020). 6G and beyond: The future of wireless communications systems. IEEE Access.

[6] Mucchi L., Jayousi S., Caputo S., Panayirci E., Shahabuddin S., Bechtold J., Morales I., Stoica R.A., Abreu G., Haas H. (2021). Physical-layer security in 6G networks. IEEE Open J. Commun. Soc..

[7] Xie N., Li Z., Tan H. (2020). A survey of physical-layer authentication in wireless communications. IEEE Commun. Surv. Tutor..

[8] Wen Y., Huo Y., Ma L., Jing T., Gao Q. (2019). A scheme for trustworthy friendly jammer selection in cooperative cognitive radio networks. IEEE Trans. Veh. Technol..

[9] Ye R., Peng Y., Al-Hazemi F., Boutaba R. (2023). A robust cooperative jamming scheme for secure UAV communication via intelligent reflecting surface. IEEE Trans. Commun..

[10] Zheng T.X., Yang Z., Wang C., Li Z., Yuan J., Guan X. (2021). Wireless covert communications aided by distributed cooperative jamming over slow fading channels. IEEE Trans. Wirel. Commun..

[11] Hong S., Pan C., Ren H., Wang K., Nallanathan A. (2020). Artificial-noise-aided secure MIMO wireless communications via intelligent reflecting surface. IEEE Trans. Commun..

[12] Wen Y., Liu L., Li J., Li Y., Wang K., Yu S., Guizani M. (2024). Covert communications aided by cooperative jamming in overlay cognitive radio networks. IEEE Trans. Mob. Comput..

[13] Wen Y., Jing T., Gao Q. (2021). Trustworthy Jammer Selection with Truth-Telling for Wireless Cooperative Systems. Wirel. Commun. Mob. Comput..

[14] Wen Y., Wang F., Wang H.M., Li J., Qian J., Wang K., Wang H. (2024). Cooperative Jamming Aided Secure Communication for RIS Enabled Symbiotic Radio Systems. IEEE Trans. Commun..

[15] Yang L., Yang J., Xie W., Hasna M.O., Tsiftsis T., Di Renzo M. (2020). Secrecy performance analysis of RIS-aided wireless communication systems. IEEE Trans. Veh. Technol..

[16] Mu X., Liu Y., Guo L., Lin J., Schober R. (2021). Simultaneously transmitting and reflecting (STAR) RIS aided wireless communications. IEEE Trans. Wirel. Commun..

[17] Pogaku A.C., Do D.T., Lee B.M., Nguyen N.D. (2022). UAV-assisted RIS for future wireless communications: A survey on optimization and performance analysis. IEEE Access.

[18] Cao X., Başar T. (2022). Distributed constrained online convex optimization over multiple access fading channels. IEEE Trans. Signal Process..

[19] Amiriara H., Ashtiani F., Mirmohseni M., Nasiri-Kenari M. (2023). Irs-user association in irs-aided miso wireless networks: Convex optimization and machine learning approaches. IEEE Trans. Veh. Technol..

[20] Feng K., Wang Q., Li X., Wen C.K. (2020). Deep reinforcement learning based intelligent reflecting surface optimization for MISO communication systems. IEEE Wirel. Commun. Lett..

[21] Lu X., Xiao L., Dai C., Dai H. (2020). UAV-aided cellular communications with deep reinforcement learning against jamming. IEEE Wirel. Commun..

[22] Feriani A., Hossain E. (2021). Single and multi-agent deep reinforcement learning for AI-enabled wireless networks: A tutorial. IEEE Commun. Surv. Tutor..

[23] Hu S., Chen X., Ni W., Hossain E., Wang X. (2021). Distributed machine learning for wireless communication networks: Techniques, architectures, and applications. IEEE Commun. Surv. Tutor..

[24] Huo Y., Wu Y., Li R., Gao Q., Luo X. (2021). A learning-aided intermittent cooperative jamming scheme for nonslotted wireless transmission in an IoT system. IEEE Internet Things J..

[25] Tusha A., Arslan H. (2024). Interference burden in wireless communications: A comprehensive survey from PHY layer perspective. IEEE Commun. Surv. Tutor..

[26] Dai L., Huang H., Zhang C., Qiu K. (2023). Silent flickering RIS aided covert attacks via intermittent cooperative jamming. IEEE Wirel. Commun. Lett..

[27] Arzykulov S., Celik A., Nauryzbayev G., Eltawil A.M. (2023). Artificial noise and RIS-aided physical layer security: Optimal RIS partitioning and power control. IEEE Wirel. Commun. Lett..

[28] Zhao B., Wu J., Ma Y., Yang C. (2024). Meta-learning for wireless communications: A survey and a comparison to gnns. IEEE Open J. Commun. Soc..

[29] Cai C., Yuan X., Zhang Y.J.A. (2023). RIS partitioning based scalable beamforming design for large-scale MIMO: Asymptotic analysis and optimization. IEEE Trans. Wirel. Commun..

[30] Chen P., Li X., Matthaiou M., Jin S. (2023). DRL-based RIS phase shift design for OFDM communication systems. IEEE Wirel. Commun. Lett..

[31] Wen Y., Liu L., Li J., Hou X., Zhang N., Dong M., Atiquzzaman M., Wang K., Huo Y. (2023). A covert jamming scheme against an intelligent eavesdropper in cooperative cognitive radio networks. IEEE Trans. Veh. Technol..

[32] Li X., Jiang J., Wang H., Han C., Chen G., Du J., Hu C., Mumtaz S. (2023). Physical layer security for wireless-powered ambient backscatter cooperative communication networks. IEEE Trans. Cogn. Commun. Netw..

[33] Su N., Liu F., Masouros C. (2023). Sensing-assisted eavesdropper estimation: An ISAC breakthrough in physical layer security. IEEE Trans. Wirel. Commun..

[34] Saggese F., Croisfelt V., Kotaba R., Stylianopoulos K., Alexandropoulos G.C., Popovski P. (2024). On the impact of control signaling in RIS-empowered wireless communications. IEEE Open J. Commun. Soc..

[35] Chapala V.K., Zafaruddin S.M. (2023). Intelligent connectivity through RIS-assisted wireless communication: Exact performance analysis with phase errors and mobility. IEEE Trans. Intell. Veh..

[36] Liang J.C., Zhang L., Luo Z., Jiang R.Z., Cheng Z.W., Wang S.R., Sun M.K., Jin S., Cheng Q., Cui T.J. (2024). A filtering reconfigurable intelligent surface for interference-free wireless communications. Nat. Commun..

[37] Aung P.S., Park Y.M., Tun Y.K., Han Z., Hong C.S. (2023). Energy-efficient communication networks via multiple aerial reconfigurable intelligent surfaces: DRL and optimization approach. IEEE Trans. Veh. Technol..

[38] Zhang S., Bao S., Chi K., Yu K., Mumtaz S. (2023). DRL-based computation rate maximization for wireless powered multi-AP edge computing. IEEE Trans. Commun..

[39] Luo Z.-Q., Ma W.-K., So A.M.-C., Ye Y., Zhang S. (2010). Semidefinite relaxation of quadratic optimization problems. IEEE Signal Process. Mag..

[40] Ahmed M., Raza S., Soofi A.A., Khan F., Khan W.U., Abideen S.Z.U., Xu F., Han Z. (2024). Active reconfigurable intelligent surfaces: Expanding the frontiers of wireless communication-a survey. IEEE Commun. Surv. Tutor..

